# Phosphorus containing analogues of SAHA as inhibitors of HDACs

**DOI:** 10.1080/14756366.2022.2063281

**Published:** 2022-05-05

**Authors:** Michael D. Pun, Hsin-Hua Wu, Feyisola P. Olatunji, Britany N. Kesic, John W. Peters, Clifford E. Berkman

**Affiliations:** a Department of Chemistry, Washington State University, Pullman, WA, USA; b Institute of Biological Chemistry, Washington State University, Pullman, WA, USA

**Keywords:** HDAC, phosphate, phosphoramidate, phosphorothiolate, SAHA

## Abstract

Histone deacetylases (HDACs) are a family of enzymes responsible for regulating DNA transcription by modulating its binding to histone proteins. HDACs are overexpressed in several types of cancers and are recognised as drug targets. Vorinostat, or suberanilohydroxamic acid (SAHA), is an histone deacetylase (HDAC) inhibitor with a hydroxamic acid as a zinc-binding group (ZBG), and it has been FDA approved for the treatment of T-cell lymphoma. In this work, phosphorus-based SAHA analogues were synthesised to assess their zinc-binding effectiveness compared to the hydroxamic acid of SAHA. Specifically, we examined phosphate, phosphoramidate and phosphorothiolate groups as isosteres of the canonical hydroxamic acid motif of conventional HDAC inhibitors. The compounds were screened for binding to HDAC enzymes from HeLa cell lysate. The most potent derivatives were then screened against HDAC3 and HDAC8 isoforms. HDAC inhibition assays demonstrated that these phosphorus-based SAHA analogs exhibited slow binding to HDACs but with greater potency than phosphonate SAHA analogs examined previously. All compounds inhibited HDACs, the most potent having an IC_50_ of 50 µM.

## Graphical Abstract

**Figure:**
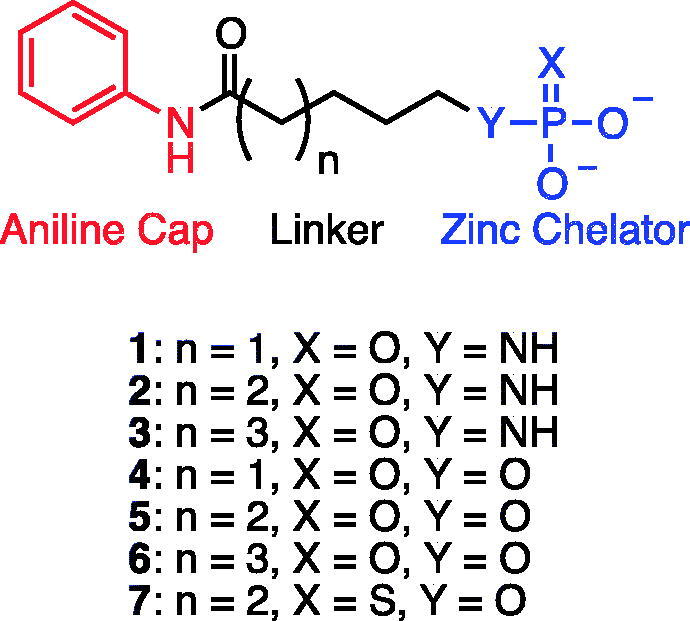

## Introduction

Chromatin is a protein–DNA complex that consist of segments of DNA wrapped around a histone octamer which are then woven into fibres^1^^,^^2^. These chromatin fibres condense the vast amounts of DNA into compact dense structures^3^^,^^4^. Histones proteins are modified via acetylation or deacetylation by histone acetyltransferase HAT and histone deacetylase HDAC enzymes, respectively^5^^,^^6^ to regulate DNA transcription by affecting how tightly DNA strands are bound to histone proteins^7^. HDACs inhibit transcription by removing *N-*acetyl modifications on histone lysine residues allowing the histone to carry a positive charge and thereby strengthening its electrostatic interactions with DNA^8^^,^^9^.

The HDAC family of zinc metalloproteinases contains 11 members and are conserved across all eukaryotes^10^. With the exception of NAD^+^-dependent class III HDACS, all HDAC family enzymes share a common catalytic mechanism. In brief, a zinc (II) ion in the active site functions to simultaneously coordinate a water molecule and act as a Lewis acid towards substrate acetyl groups^11^. This coordination serves to lower the p*K*a of the water molecule and polarise the carbonyl group, thus increasing the nucleophilicity and electrophilicity of each, respectively^12^. Nucleophilic addition of water to the carbonyl centre of the substrate acetyl leads to a tetrahedral intermediate^13^, which, once collapsed, releases the lysine amine and acetic acid.

HDACs have served as drug targets for many diseases including various cancers, interstitial fibrosis, autoimmune and inflammatory diseases, and metabolic disorders^14^. Indeed, considerable efforts have been made to develop HDAC inhibitors (HDACi). Vorinostat, or SAHA, is a broad spectrum HDACi (IC_50_ = 13 nM) has been FDA approved to treat cutaneous T-cell lymphoma^15^^,^^16^. This molecule utilises a hydroxamic acid as a zinc-binding group^17^ as do Belinostat and Panobinostat^18^, while a thiol serves as the zinc-binding group in Romidepsin^19^. Because none of the known HDAC inhibitors are specific for a single HDAC, off-target effects remain an issue.

In recent work, much attention has been towards increasing the potency of HDACi molecules and improving selectivity for certain isoenzymes. Negmeldin et al. modified the C2 position of SAHA with a n-hexyl to exploit a wider active site entrance of HDAC6/8. This compound resulted in a 49- to 300-fold HDAC6/8 (IC50 = 0.6 and 2.0 µM) selectivity over HDAC1-3^20^. Procainamide-SAHA fused inhibitors proposed by Nardella et al. targeted post translational modifications in the malaria parasite plasmodium falciparum. This compound combines SAHA, a potent pan-HDAC inhibitor with a DNA methyltransferase inhibitor procainamide. The lead SAHA/procainamide fusion molecule was fully active in drug resistant plasmodium falciparum isolates (IC50 = 41 nM) and human HDAC6 (IC50 = 14 nM)^21^. Another strategy for optimisation of SAHA derivatives is replacing the anilide with different hydrophobic functional groups. Huang et al. synthesised and evaluated SAHA derivatives with osthole fused to the aliphatic hydroxamate core. Their best compound showed potency and selectivity similar to SAHA with moderate selectivity towards HDAC6 (IC50 = 14 nM)^22^.

**Figure 1.**
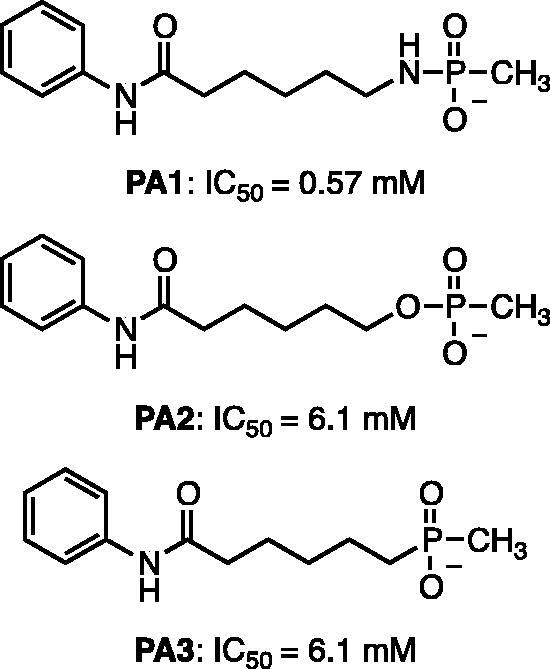
Phosphorus containing SAHA analogues discovered by Kapustin et al. IC_50_ values reported for Hela cell lysate and 10 h incubation time.

Kapustin et al.^23^ demonstrated the utility of phosphonamidate, phosphonate and phosphinate analogs of SAHA (Figure 1) as HDAC inhibitors. The most potent of these was a monobasic phosphoramidate-based compound **PA1** (Figure 1) with an IC_50_ of 570 µM against HeLa cell lysates. It also exhibited a slow binding mode of inhibition, requiring a 10-h preincubation time. The focus of this study was to expand upon the Kapustin study by examining dibasic phosphoryl motifs as zinc-binding groups in the context of HDAC inhibitors.

Our inhibitor selectivity experiments focussed on the Class-I HDACs (HDAC1, 2, 3, and 8). The HDAC isoforms 3 and 8 were chosen from this class due to the differences in their sequence and structure. Both of these enzymes are present in the cell nucleus and use zinc as a cofactor for catalytic activity. There are 4 key differences in the active site amino acid sequence suggesting that there is a selectivity towards substrates^24^. HDAC8 contains a flexible L1 loop made up of 7 amino acids that form a hydrophobic secondary pocket adjacent to the active site^25^. This pocket has been exploited for HDAC8-specific inhibitor research and has led to “L shaped” molecules with improved activity against HDAC8^26^. These HDACs are clinically relevant due to HDAC 8 being overexpressed in T-Cell leukaemia and Neuroblastoma^27^^,^^28^. HDAC3, however, is associated with neurodegenerative diseases such as Alzheimer’s disease.

The time course enzyme inhibition assay using compound **2** showed optimal inhibition at 8 h for HeLa cell Lysate and HDAC 8, and 4 h for HDAC 3. These results suggest that our series of phosphoryl compounds are slow binding inhibitors. These types of inhibitors also express tight binding qualities such that the molecules have low dissociation rates and long drug target residence time^29^. In vitro, this strong binding quality can disrupt cell viability due to the inhibitors ability to shutdown the enzyme for a long period of time. If enzyme synthesis time in targeted cells cannot overcome the inhibition time, cell viability can be affected^30^. The advantage of slow and tight binding inhibitors for in vivo biomedical purposes stems from the decreased off target toxicity of the compound. Because of the decreased systemic circulation time and increased inhibitor residence time a lower concentration of the compound is available in the blood stream to bind to non-targeted protein^31^. There are several known examples of slow binding as FDA-approved drugs^32^. There are also several know types of HDAC inhibitors that exhibit slow binding kinetics^33^.

## Results and discussion

**Scheme 1.**
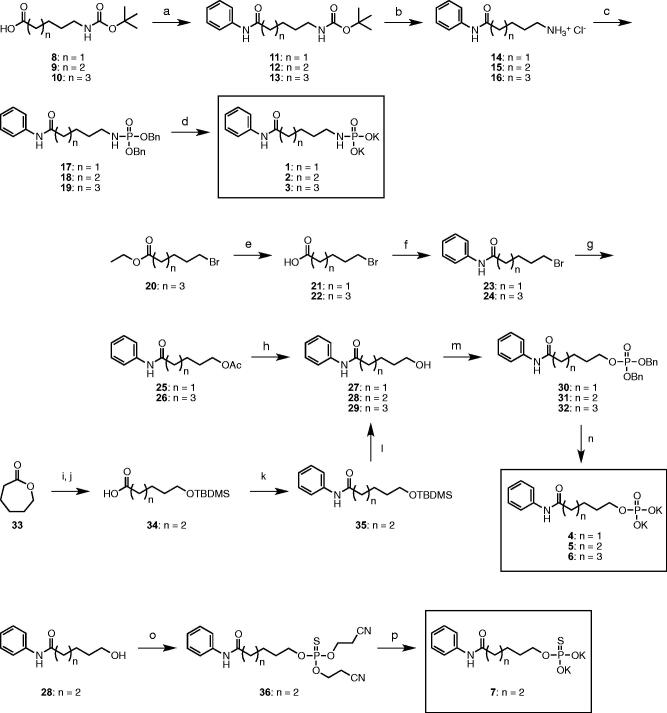
Synthesis of HDAC inhibitors **1**–**7**. a) HBTU, DIPEA, aniline, DMF b) 4 N HCl in dioxane c) dibenzyl phosphite, BrCCl_3_, Et_3_N, CH_3_CN d) H_2_, Pd/C, MeOH, KHCO_3_ e) 33% HBr in AcOH f) DCC, DMAP, aniline, DMF g) KOAc, DMF, 70 °C h) NaOH, MeOH i) NaOH, H_2_O j) imidazole, TBDMS-Cl, DCM k) HBTU, DIPEA, aniline, DMF l) 4 N HCl in dioxane m) Dibenzyl *N,N*-diisopropylphosphoramidite, 5-(Ethylthio)-1*H*-tetrazole, ACN, *tert*-butyl hydrogen peroxide n) H_2_, Pd/C, MeOH, KHCO_3_ o) Bis(2-cyanoethyl)-*N,N*-diisopropylphosphoramidite, Sulphur, ACN p) KOH, MeOH.

Amino aniline amides^14–16^ were synthesised from commercially available Boc-protected amino acids by a HBTU coupling reaction with aniline followed by deprotection with HCl (Scheme 1). Phosphoramidates^17–19^ were synthesised by an Atherton-Todd reaction with amino aniline amides and dibenzylphosphite. The resulting dibenzyl protected phosphoramidates were deprotected by catalytic hydrogenation in the presence of potassium bicarbonate to provide products^1–3^. Hydroxy aniline amides^27–29^ were synthesised either starting from the commercially available bromo-alkyl ester or corresponding lactone. Ethyl 7-bromoheptanoate **20** was hydrolysed using HBr in acetic acid to provide 7-bromoheptanoic acid **22**. Both bromoheptanoic acid **22** and commercially available bromopentanoic acid **21** were coupled with aniline using DCC. An O-acetyl group was installed by reaction with bromo alkyl acid **23** and **24** and potassium acetate. Saponification with NaOH was preformed to provide the alcohols **27** and **29**. ε-Caprolactone was hydrolysed and TBDMS protected following an established literature procedure (ref). TBDMS protected alkyl acid was coupled to aniline using HBTU followed by deprotection of the silyl ester using aqueous acid to provide alcohol **28** (34). The alcohols were treated with dibenzyl diisopropylphosphoramidite and oxidised with *tert-*butyl hydrogen peroxide to give the dibenzyl phosphates^30–32^. Deprotection was accomplished by catalytic hydrogenation in the presence of potassium bicarbonate to provide products^4–6^. Phosphorothioates were synthesised by reacting 6-hydroxy-*N*-phenylhexamadmide **28** with bis(2-cyanoethyl) diisopropylphosphoramidite then oxidising with elemental sulphur. The cyanoethyl esters were removed by treating with an excess of sodium hydroxide to give product **7**.

**Figure 2.**
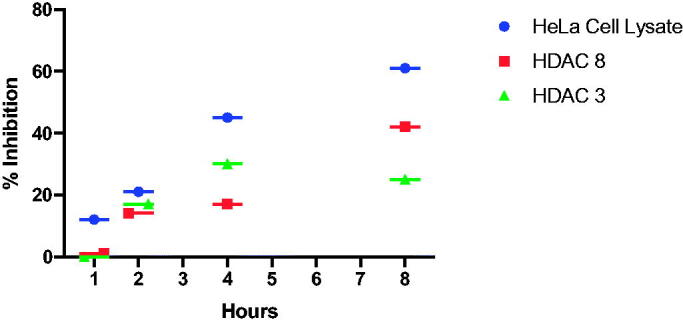
Time-dependent inhibition of HDACs from HeLa cell lysates, HDAC8 and HDAC3 with inhibitor **2 (**100 μM).

Once prepared, the compounds were screened for inhibition of HDAC activity from the cell lysates of HeLa cells and the recombinant isoforms HDAC3 and HDAC8. Linker lengths of 5–7 atoms (including O or NH) were chosen for these structures to compare to the analogous structure of SAHA with its 6-atom linker between the hydrophobic analide cap and hydroxamate ZBG. While incubation times less than 1 h resulted in little inhibition of HDAC activity, the compounds exhibited significant inhibition when pre-incubated with HeLa cell lysates, for 8 h (Figure 2), which was consistent with phosphonyl-based HDAC inhibitors (**PA1-3**). Recombinant HDAC3 and HDAC8 were also tested for slow binding inhibition using compound 2.

The screening results (Table 1) showed evidence of concentration dependent inhibition for each compound. The most potent compounds were the phosphoramidate **2** and phosphate **5**, both possessing a 6-atom linker. Based on these results, we further expanded the library to include the thiophosphate **7**, while maintaining a 6-atom linker. Interestingly, the inhibitory potency of compounds **2**, **5**, and **7** were similar, suggesting little difference in the zinc-binding of these the three motifs; phosphoramidate, phosphate, and thiophosphate. Therefore, we tested these compounds **2**, **5**, and **7** using recombinant HDAC3 and HDAC8 (Table 1). The assay results showed that compound **2** had the highest potency for the cell lysate (IC50 = 70 ± 8 µM) but was more selective for HDAC8 (IC50 = 129 ± 23 µM) over HDAC3 (IC50 = 240 ± 34 µM). Similarly compound **5** also had the highest potency for cell lysate (IC50 = 60 ± 9 µM), and was more selective for HDAC8 (IC50 = 179 ± 34 µM) over HDAC3 (IC50 = 690 ± 60 µM). Compound **7** showed similar potency between cell lysate (IC50 = 50 ± 13 µM) and HDAC8 (IC50 = 49 ± 8 µM) but was less selective for HDAC3 (IC50 = 103 ± 20 µM).

**Table 1.** IC_50_ values for inhibitors **1**–**7** and **SAHA**.

Compound	Structure	HeLa Cell Lysate IC50 (μM)^a^	Recombinant HDAC 8 IC50 (μM)^a^	Recombinant HDAC 3 IC50 (μM)^a^
**1**	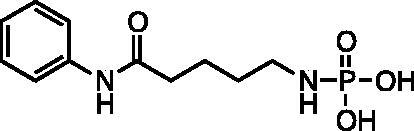	360 (60)	NT	NT
**2**	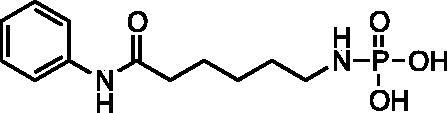	70 (8)	129 (23)	240 (34)
**3**	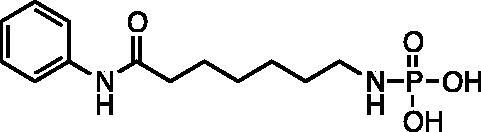	490 (9)	NT	NT
**4**	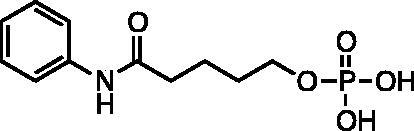	2200 (570)	NT	NT
**5**	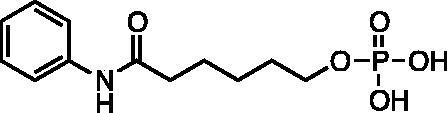	60 (9)	179 (34)	690 (60)
**6**	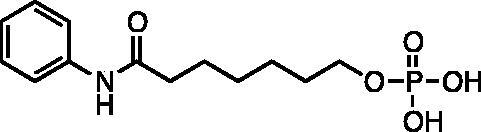	3900 (1800)	NT	NT
**7**	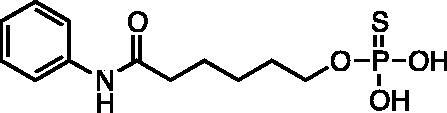	50 (13)	49 (8)	103 (20)
**SAHA**	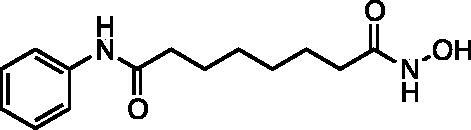	0.2 (0.04)	1 (0.3)	0.24 (0.02)

^a^Determined for HeLa cell extract, recombinant HDAC3 and HDAC8 using a fluorometric assay incubating for 8 h. Standard deviation in parentheses. NT = Not Tested.

## Conclusion

In summary, we synthesised a library of 7 phosphoryl-based analogs of SAHA. These inhibitors were designed to contain the canonical hydrophobic anilide cap and aliphatic linker, but present an alternative phosphoryl-based ZBG. While each of these motifs could provide multidentate interactions with the HDAC active-site zinc ion, this was not an advantage with respect to inhibitory potency against HDACs. However, while these compounds were considerably less potent than SAHA (0.2 µM) against HDACs from HeLa cell lysates or HDAC3 (0.24 µM) and HDAC8 (1 µM), they exhibited greater potency compared to previously reported phopshonyl-based HDAC inhibitors (23). Investigations into the specificity of inhibitory potency against individual HDACs and cancer cells by these compounds will be forthcoming.

## Supplementary Material

Supplemental Material
