# Supplementary material for: Phosphorus containing analogues of SAHA as inhibitors of HDACs
Source: J Enzyme Inhib Med Chem. 2022 May 5;37(1):1315–9. doi: 10.1080/14756366.2022.2063281 (PMC9090410; doi:10.1080/14756366.2022.2063281)
Supplement: Supplemental Material [file IENZ_A_2063281_SM3122.pdf]

General. *In Situ* Histone Deacetylase Assay Kit, Fluorometric and SAHA were purchased from Sigma-Aldrich (St. Louis, MO). All other chemical and biological reagents were purchased from Fisher Scientific (Sommerville, NJ), Sigma-Aldrich (St. Louis, MO) or Chem-Impex International (Wood Dale, IL). All solvents used in reactions were anhydrous and obtained from chemical sources or distilled prior to use. All other reagents were used as received without further purification. Silica gel flash chromatography was carried out using a Biotage Flash Plus chromatography system. ^1H and ^{13}C NMR spectra were recorded on a 300, 400 or 600-MHz Varian NMR spectrometer. ^1H NMR chemical shifts are relative to $CDCl_3$ (δ = 7.26 ppm), D_2O (δ = 4.79 ppm), or MeOD (δ = 3.31 ppm). ^{13}C NMR chemical shifts are relative to $CDCl_3$ (δ = 77.16 ppm) or MeOD (δ = 49.00 ppm). High-resolution mass spectrometry was performed using an ABI 4800 MALDI TOF/TOF Analyzer (Applied Biosystems), Synapt G2-S Q-TOF (Waters) mass spectrometer with an Acquity UPLC system and photodiode array detector, or 6530 series Q-TOF MS with ESI source (Agilent). Fluorometric assay was preformed using a BMG CLARIOstar plate reader.

Chemical Synthesis.

General procedure for HBTU aniline coupling for compounds: 11, 12, 13 & 35. To a 20 mL scintillation vial with stir bar was added 300 mg of carboxylic acid (**8-10 & 34**), HBTU (1.5 equiv.), DIPEA (2.5 equiv.) and DMF (3 mL) the mixture was allowed to stir at 0 ^oC for 15 min. Then aniline (2 equiv.) was added and the reaction was capped under argon and allowed to stir overnight. The reaction mixture was diluted using 50 mL of EtOAc and washed with 3 x 10 mL each of 10% HCl, saturated aqueous $NaHCO_3$ and 1 x 10 mL of brine. The organic layer was collected and treated with $NaSO_4$ and dried *in vacuo*. The crude was purified by SiO_2 column chromatography to provide the title compounds as a colorless oil.

General procedure for DCC aniline coupling for compounds: 23 & 24. To a 20 mL scintillation vial was added Bromo alkyl carboxylic acid **21 &22** (300 mg) in DCM (2 mL). Then DCC (1.2 equiv.) was added followed by aniline (1.5 equiv.) was added. The vial was capped and reaction was allowed to proceed overnight. Next the solid precipitate was removed by filtration and the resulting mixture was purified by column chromatography to provide title compounds a colorless oil.

General procedure for acetylation to provide compounds: 25 & 26. To a 20 mL scintillation vial with stir bar was added bromo alkyl anilide **23 & 24** (200 mg) in DMF (2 mL) followed by potassium acetate (3 equiv.). The reaction mixture was heated to 70 ^{o}C and allowed to react overnight. Crude mixture was then filtered and diluted to 50 mL of ethyl acetate, organic layer was washed with 3 x 20 mL of water then 20 mL of brine. Organic layer was collected treated with Na_2SO_4 and dried in vacuo. Next crude was purified by column chromatography to provide product as a colorless oil.

General procedure for saponification to provide compounds: 27 & 29. To a 20 mL scintillation vial with stir bar was added a solution of acetyl alkyl anilide **25 & 26** (150 mg) in dioxane (5 mL). Next a solution of NaOH (3 equiv.) in water (2 mL) was added and reaction was stirred at room temperature overnight. Solution was then neutralized with aqueous 1N HCl and extracted with 3 x 15 mL of EtOAc. Organic layer were combined and treated with Na_2SO_4 and dried *in vacuo* to provide title compounds used without further purification.

General procedure for Boc deprotections for compounds: 14, 15 & 16. To a 20 mL scintillation vial containing 200 mg carbamate (**11-13**) was added 3 mL of 4.0 N HCl in dioxane. The vial was capped and allowed to sit overnight, then solvent was removed in vacuo to provide title compounds used without further purification.

General procedure for Atherton Todd reaction to produce compounds: 17, 18 & 19. To a flame dried 50 mL round bottom flask with stir bar was added dibenzylphosphite (50 mg, 1 equiv.), 15 mL of $BrCCl_3$, 15 mL of anhydrous acetonitrile, and pyridine (4 equiv.), solution was then placed under argon atmosphere and cooled to -20 ^oC. Amine (**14-16**) (2 equiv.) was dissolved in acetonitrile and pyridine (2 equiv.) and was dropwise to the dibenzylphosphite solution. Reaction was allowed to react for 2 hours at -20 ^oC then warm to room temperature overnight. Then reaction was filtered, concentrated in vacuo and purified by column chromatography to provide title compounds as a colorless oil.

General procedure for production of dibenzyl phosphates: 30, 31 & 32. To a flame dried 50 mL round bottom flask with stir bar under argon atmosphere was added dibenzyl N,N-diisopropylphosphoramidite, (100 mg, 1 equiv.) in acetonitrile. Solution was cooled to 0 ^oC, then 5-(ethyl)-1H-tetrazole (1.2 equiv.) was added and the mixture was allowed to stir for 30 min. A solution of alcohol (**29-31**) (1.5 equiv.) in acetonitrile was added dropwise to the phosphoramidite. After 2 hours tert-butyl hydrogen peroxide (2 equiv.) was added then reaction was stirred overnight. Mixture was filtered, concentrated and residue was purified by column chromatography to provide title compounds as a colorless oil.

General procedure for hydrogenolysis of dibenzyl phosphorus compounds: 1-6. To 5 mL round bottom flask with stir bar was added 50 mg of dibenzyl phosphoramidate (**17-19**) or dibenzyl phosphate (**32-34**) in methanol (2 mL) then 10% palladium on carbon (0.4 equiv.) and $KHCO_3$ (2 equiv.) was added. The flask was sealed with a septum, flushed with argon and evacuated. A balloon of hydrogen gas was added and reaction was stirred overnight. Then balloon was removed and flask was evaporated and filled with argon. Mixture was then filtered and solvent removed to provide title compound as a white solid.

Potassium (5-oxo-5-(phenylamino)pentyl)phosphoramidate (**1**). Yield: 98%, 37.5 mg. ^{1}H NMR (400 MHz, Deuterium Oxide) δ 7.39 – 7.27 (m, 4H), 7.18 (p, *J* = 4.4 Hz, 1H), 2.89 (td, *J* = 7.3, 5.1 Hz, 2H), 2.38 (t, *J* = 7.2 Hz, 2H), 1.73 – 1.52 (m, 4H). ^{13}C NMR (101 MHz, D_2O) δ 175.45, 136.69, 129.18, 125.64, 122.19, 42.50, 35.88, 28.13, 22.73. ^{31}P NMR (162 MHz, D_2O) δ 3.52. HRMS (MALDI): m/z calculated for $C_{11}H_{17}N_2O_4P$ [M+H] 273.09259; observed 273.09979.

Potassium (6-oxo-6-(phenylamino)hexyl)phosphoramidate (**2**). Yield: 97%, 38 mg. ^{1}H NMR (400 MHz, Deuterium Oxide) δ 7.32 (d, *J* = 4.4 Hz, 4H), 7.15 (p, *J* = 4.6 Hz, 1H), 2.80 (tdd, *J* = 7.6, 5.6, 1.9 Hz, 2H), 2.32 (t, *J* = 7.4 Hz, 2H), 1.61 (p, *J* = 7.5 Hz, 2H), 1.55 – 1.45 (m, 2H), 1.32 (p, *J* = 7.7 Hz, 2H). ^{13}C NMR (101 MHz, D_2O) δ 175.86, 136.72, 129.15, 125.58, 122.10, 42.62, 36.28, 29.10, 25.75, 25.08. ^{31}P NMR (162 MHz, D_2O) δ 5.12. HRMS (ESI): m/z calculated for $C_{12}H_{19}N_2O_4P$ [M-H] 285.1082; observed 285.1005.

Potassium (7-oxo-7-(phenylamino)heptyl)phosphoramidate (**3**). Yield: 97%, 31 mg. ^{1}H NMR (600 MHz, Deuterium Oxide) δ 7.57 – 7.44 (m, 4H), 7.33 (d, *J* = 5.5 Hz, 1H), 3.15 – 3.04 (m, 2H), 2.50 (td, *J* = 8.2, 7.7, 2.7 Hz, 2H), 1.76 (d, *J* = 27.3 Hz, 4H), 1.56 – 1.42 (m, 4H). ^{13}C NMR (151 MHz, D_2O) δ 176.05, 136.72, 129.25, 125.74, 122.27, 39.52, 36.29, 27.64, 26.54, 25.30, 25.04. ^{31}P NMR (243 MHz, D_2O) δ 0.69. HRMS (MALDI): m/z calculated for $C_{13}H_{21}N_2O_4P$ [M+H] 301.12389; observed 301.13181.

Potassium 5-oxo-5-(phenylamino)pentyl phosphate (**4**). Yield: 99%, 38 mg. ^{1}H NMR (600 MHz, Deuterium Oxide) δ 7.31 – 7.25 (m, 4H), 7.11 (tt, *J* = 5.2, 3.2 Hz, 1H), 3.65 (td, *J* = 6.5, 5.6 Hz, 2H), 2.32 (t, *J* = 7.5 Hz, 2H), 1.70-1.62 (m, 2H), 1.58 – 1.47 (m, 2H). ^{13}C NMR (151 MHz, D_2O) δ 175.87, 136.73, 129.12, 125.55, 122.17, 63.71, 63.68, 36.06, 29.52, 29.47, 21.98. ^{31}P NMR (243 MHz, D_2O) δ 3.89. HRMS (MALDI): m/z calculated for $C_{11}H_{16}NO_5P$ [M+K] 274.07661; observed 274.08478.

Potassium 6-oxo-6-(phenylamino)hexyl phosphate (**5**). Yield: 98%, 38 mg. ^{1}H NMR (600 MHz, Deuterium Oxide) δ 7.26 (d, *J* = 4.4 Hz, 4H), 7.12 – 7.06 (m, 1H), 3.61 (td, *J* = 6.9, 5.7 Hz, 2H), 2.26 (t, *J* = 7.5 Hz, 2H), 1.56 (p, *J* = 7.5 Hz, 2H), 1.49 (p, *J* = 7.1 Hz, 2H), 1.32 – 1.24 (m, 2H). ^{13}C NMR (151 MHz, D_2O) δ 175.96, 136.77, 129.12, 125.51, 122.07, 64.28, 64.25, 36.39, 30.22, 29.92, 29.88, 25.17, 24.68. ^{31}P NMR (243 MHz, D_2O) δ 3.71. HRMS (MALDI): m/z calculated for $C_{12}H_{18}NO_5P$ [M+H] 288.09226; observed 288.10074.

Potassium 7-oxo-7-(phenylamino)heptyl phosphate (**6**). Yield: 99%, 39 mg. ^{1}H NMR (600 MHz, Deuterium Oxide) δ 7.30 – 7.24 (m, 4H), 7.13 – 7.06 (m, 1H), 3.64 – 3.58 (m, 2H), 2.25 (t, *J* = 7.4 Hz, 2H), 1.54 (dd, *J* = 8.5, 5.5 Hz, 2H), 1.50 – 1.42 (m, 2H), 1.25 (p, *J* = 3.6 Hz, 4H). ^{13}C

NMR (151 MHz, D_2O) δ 176.06, 136.76, 129.13, 125.52, 122.08, 64.60, 64.57, 36.36, 30.11, 30.06, 28.04, 25.27, 24.77. ^{31}P NMR (243 MHz, D_2O) δ 3.53. HRMS (MALDI): m/z calculated for $C_{13}H_{20}NO_5P$ [M+H] 302.10791; observed 302.11673.

Potassium O-(6-oxo-6-(Phenylamino)hexyl) phosphorothioate (**7**). To a stirring solution of dicyanotheyl ester (**36**) (55 mg, 0.114 mmol) in methanol (2 mL) was added a solution of KOH (13 mg, 0.228 mmol, 2 equiv.) in water (1 mL). Reaction was stirred for 20 h at room temperature then filtered and lyophilized to a white powder used in the next step without further purification (42 mg, 98 % yield). ^{1}H NMR (400 MHz, Deuterium Oxide) δ 7.33 (t, *J* = 4.7 Hz, 4H), 7.23 – 7.09 (m, 1H), 3.87 (dt, *J* = 7.9, 6.5 Hz, 2H), 2.36 (t, *J* = 7.4 Hz, 2H), 1.64 (dp, *J* = 13.9, 7.0, 6.6 Hz, 4H), 1.39 (tt, *J* = 9.3, 6.0 Hz, 2H). ^{31}P NMR (162 MHz, D_2O) δ 51.39. HRMS (ESI): m/z calculated for $C_{12}H_{18}NO_4PS$ [M-H] 302.0694; observed 302.0621.

tert-butyl (5-oxo-5-(Phenylamino)pentyl)carbamate (**11**). Yield: 99%, 400 mg. ^{1}H NMR (400 MHz, Methanol-d_4) δ 7.62 – 7.51 (m, 2H), 7.36 – 7.24 (m, 2H), 7.08 (td, *J* = 7.3, 1.3 Hz, 1H), 3.09 (td, *J* = 7.0, 5.1 Hz, 2H), 2.39 (t, *J* = 7.5 Hz, 2H), 1.71 (tt, *J* = 7.7, 6.1 Hz, 2H), 1.54 (p, *J* = 7.0 Hz, 2H), 1.43 (s, 9H). ^{13}C NMR (101 MHz, MeOD) δ 173.00, 157.18, 138.44, 128.44, 123.83, 119.96, 78.57, 39.62, 36.19, 29.15, 27.52, 22.75.

tert-butyl (6-oxo-6-(Phenylamino)hexyl)carbamate (**12**). Yield: 99%, 395 mg. ^{1}H NMR (400 MHz, Methanol-d_4) δ 9.88, 7.64 (d, *J* = 8.0 Hz, 2H), 7.32 (t, *J* = 7.7 Hz, 2H), 7.06 (t, *J* = 7.4 Hz, 1H), 6.81 (t, *J* = 5.8 Hz, 1H), 2.96 (q, *J* = 6.6 Hz, 2H), 2.34 (t, *J* = 7.4 Hz, 2H), 1.63 (p, *J* =

7.5 Hz, 2H), 1.54 – 1.44 (m, 2H), 1.41 (s, 9H), 1.32 (p, J = 7.8 Hz, 2H).^{13}C NMR (101 MHz, MeOD) δ 172.46, 156.87, 140.62, 129.87, 124.16, 120.31, 78.57, 37.65, 30.62, 29.53, 27.29, 26.16.

tert-butyl (7-oxo-7-(Phenylamino)heptyl)carbamate (**13**). Yield: 98%, 385 mg. ^{1}H NMR (400 MHz, Methanol-d_4) δ 8.44 – 8.36 (m, 2H), 8.13 – 8.04 (m, 2H), 7.82 (tt, J = 7.4, 1.2 Hz, 1H), 7.56 (t, J = 5.7 Hz, 1H), 3.71 (q, J = 6.6 Hz, 2H), 3.10 (t, J = 7.5 Hz, 2H), 2.39 (p, J = 7.1 Hz, 2H), 2.18 (s, 11H), 2.08 (dq, J = 8.6, 4.6 Hz, 4H). ^{13}C NMR (101 MHz, MeOD) δ 172.50, 156.86, 140.64, 129.88, 124.15, 120.30, 78.55, 37.66, 30.66, 29.70, 29.54, 27.39, 26.40.

5-oxo-5-(phenylamino)pentan-1-aminium chloride (**14**). Yield: 95%, 148 mg. ^{1}H NMR (400 MHz, Chloroform-d) δ 8.50 (s, 1H), 7.54 (d, J = 8.0 Hz, 2H), 7.28 (t, J = 7.8 Hz, 2H), 7.07 (t, J = 7.4 Hz, 1H), 2.70 (t, J = 6.9 Hz, 2H), 2.35 (t, J = 7.4 Hz, 2H), 2.19 (s, 2H), 1.73 (p, J = 7.4 Hz, 2H), 1.50 (p, J = 7.1 Hz, 2H). ^{13}C NMR (101 MHz, $CDCl_3$) δ 171.78, 138.30, 128.88, 124.07, 120.02, 41.39, 37.03, 32.41, 22.83.

6-oxo-6-(Phenylamino)hexan-1-aminium chloride (**15**). Yield: 95%, 150 mg. ^{1}H NMR (400 MHz, Deuterium Oxide) δ 7.36 – 7.28 (m, 2H), 7.22 (dd, J = 8.6, 7.2 Hz, 2H), 7.04 (td, J = 7.2, 1.3 Hz, 1H), 2.84 (t, J = 7.7 Hz, 2H), 2.23 (t, J = 7.5 Hz, 2H), 1.54 (qd, J = 8.0, 2.3 Hz, 4H), 1.26 (qd, J = 9.9, 9.1, 6.4 Hz, 2H). ^{13}C NMR (101 MHz, D_2O) δ 174.91, 137.05, 129.10, 125.23, 121.42, 66.55, 39.32, 36.21, 26.49, 25.23, 24.76.

7-oxo-7-(Phenylamino)heptan-1-aminium chloride (**16**). Yield: 99%, 159 mg. ^{1}H NMR (400 MHz, Deuterium Oxide) δ 7.33 – 7.28 (m, 2H), 7.27 – 7.15 (m, 2H), 7.10 – 6.97 (m, 1H),

2.81 (t, J = 7.6 Hz, 2H), 2.21 (t, J = 7.5 Hz, 2H), 1.58 – 1.40 (m, 4H), 1.20 (p, J = 3.7 Hz, 4H). ^{13}C NMR (101 MHz, D_2O) δ 175.24, 137.05, 129.08, 125.21, 121.40, 39.42, 36.38, 27.76, 26.55, 25.33, 25.08.

Dibenzyl (5-oxo-5-(Phenylamino)pentyl)phosphoramidate (**17**). Yield: 71%, 70 mg. ^{1}H NMR (400 MHz, Chloroform-*d*) δ 8.12 (s, 1H), 7.53 – 7.46 (m, 2H), 7.28 – 7.13 (m, 12H), 7.01 – 6.93 (m, 1H), 4.93 (dd, J = 7.5, 1.2 Hz, 4H), 2.78 (ddq, J = 20.2, 9.8, 6.9 Hz, 3H), 2.21 (t, J = 7.5 Hz, 2H), 2.13 (s, 1H), 1.57 (p, J = 7.1 Hz, 2H), 1.30 (p, J = 6.8 Hz, 2H), 1.24 – 1.13 (m, 5H). ^{13}C NMR (101 MHz, $CDCl_3$) δ 171.78, 138.46, 136.39, 128.89, 128.57, 128.34, 127.73, 123.94, 119.86, 67.98, 41.25, 37.25, 31.34, 28.57, 26.02, 25.40. ^{31}P NMR (162 MHz, $CDCl_3$) δ 9.59. HRMS (MALDI): m/z calculated for $C_{25}H_{29}N_2O_4P$ [M+1] 452.18649; observed 453.19427.

Dibenzyl (6-oxo-6-(Phenylamino)hexyl)phosphoramidate (**18**). Yield: 65%, 62 mg. ^{1}H NMR (400 MHz, Chloroform-*d*) δ 8.52 (s, 1H), 7.62 (d, J = 8.0 Hz, 2H), 7.44 – 7.23 (m, 12H), 7.07 (t, J = 7.4 Hz, 1H), 5.03 (dd, J = 7.6, 1.7 Hz, 4H), 3.14 (dt, J = 12.4, 6.9 Hz, 1H), 2.86 (dq, J = 13.3, 6.9 Hz, 2H), 2.31 (t, J = 7.4 Hz, 2H), 1.66 (p, J = 7.6 Hz, 2H), 1.44 (p, J = 7.1 Hz, 2H), 1.31 (p, J = 7.6 Hz, 2H). ^{13}C NMR (101 MHz, $CDCl_3$) δ 171.84, 138.56, 136.43, 136.36, 128.87, 128.59, 128.36, 127.72, 123.92, 119.95, 67.99, 41.15, 37.16, 31.25, 26.02, 25.05. ^{31}P NMR (162 MHz, $CDCl_3$) δ 9.57. HRMS (MALDI): m/z calculated for $C_{26}H_{31}N_2O_4P$ [M+1] 467.20214; observed 467.21045.

Dibenzyl (7-oxo-7-(Phenylamino)heptyl)phosphoramidate (**19**). Yield: 79%, 77 mg. ^{1}H NMR (400 MHz, Chloroform-*d*) δ 8.31 (s, 1H), 7.61 (d, J = 8.0 Hz, 2H), 7.47 – 7.20 (m, 13H),

7.08 (t, *J* = 7.4 Hz, 1H), 5.08 – 5.01 (m, 4H), 2.98 (dt, *J* = 11.7, 6.8 Hz, 1H), 2.91 – 2.79 (m, 2H), 2.33 (t, *J* = 7.4 Hz, 2H), 1.68 (p, *J* = 7.1 Hz, 2H), 1.42 (h, *J* = 6.6 Hz, 2H), 1.37 – 1.22 (m, 5H). ^{13}C NMR (101 MHz, $CDCl_3$) δ 171.83, 138.50, 136.46, 136.38, 128.88, 128.57, 128.34, 127.73, 123.92, 119.88, 67.97, 41.25, 37.23, 31.40, 28.57, 26.03, 25.41. ^{31}P NMR (162 MHz, $CDCl_3$) δ 9.71. HRMS (MALDI): m/z calculated for $C_{27}H_{33}N_2O_4P$ [M+Na] 504.21779; observed 504.20801.

7-bromoheptanoic acid (**21**). To a 20 mL scintillation vial with stir bar was added ethyl 7-bromoheptanoate (500 mg, 2.11 mmol) and hydrobromic acid in acetic acid (33%; 15 mL). The reaction mixture was stirred overnight at 100 °C then diluted with water to 80 mL and extracted 3 x 40 mL of dichloromethane. The organic layer was washed with water 3 x 100 mL and brine 50 mL, treated with Na_2SO_4 and dried *in vacuo* to provide product as a white solid (405 mg, 92%). ^{1}H NMR (400 MHz, Chloroform-*d*) δ 3.40 (t, *J* = 6.8 Hz, 2H), 2.36 (t, *J* = 7.4 Hz, 2H), 1.86 (p, *J* = 6.9 Hz, 2H), 1.65 (p, *J* = 7.5 Hz, 2H), 1.47 (dq, *J* = 9.1, 6.7 Hz, 2H), 1.38 (tt, *J* = 10.7, 4.3 Hz, 2H).

5-bromo-N-Phenylpentanamide (**23**). Yield: 93%, 349 mg. ^{1}H NMR (300 MHz, Chloroform-*d*) δ 8.87 (s, 1H), 7.53 (d, *J* = 7.9 Hz, 2H), 7.26 (t, *J* = 7.7 Hz, 2H), 7.08 (t, *J* = 7.4 Hz, 1H), 3.31 (t, *J* = 6.3 Hz, 2H), 2.34 (t, *J* = 6.5 Hz, 2H), 1.93 – 1.69 (m, 4H). ^{13}C NMR (101 MHz, $CDCl_3$) δ 170.51, 137.76, 129.12, 128.90, 124.31, 119.98, 119.67, 36.52, 33.18, 32.02, 24.02.

7-bromo-N-Phenylheptanamide (**24**). Yield: 90%, 244 mg. ^{1}H NMR (400 MHz, Chloroform-*d*) δ 7.85 (s, 1H), 7.56 – 7.49 (m, 2H), 7.28 (t, *J* = 7.8 Hz, 2H), 7.08 (t, *J* = 7.4 Hz, 1H), 3.37 (t, *J* = 6.7 Hz, 2H), 2.34 (t, *J* = 7.5 Hz, 2H), 1.82 (p, *J* = 6.9 Hz, 2H), 1.70 (p, *J* = 7.5 Hz,

2H), 1.40 (dddd, J = 31.8, 15.6, 8.7, 4.9 Hz, 4H). ^{13}C NMR (101 MHz, $CDCl_3$) δ 171.63, 138.06, 128.91, 124.18, 119.98, 77.42, 77.10, 76.79, 37.43, 33.94, 32.49, 28.31, 27.84, 25.39.

5-oxo-5-(Phenylamino)pentyl acetate (**25**). Yield: 57%, 164 mg. ^{1}H NMR (400 MHz, Chloroform-*d*) δ 7.51 (d, J = 8.0 Hz, 2H), 7.31 (t, J = 7.8 Hz, 2H), 7.10 (t, J = 7.4 Hz, 1H), 4.11 (dd, J = 7.7, 4.7 Hz, 2H), 2.39 (t, J = 7.2 Hz, 2H), 1.77 – 1.82 (m, 2H), 1.75 – 1.70 (m, 2H). ^{13}C NMR (101 MHz, $CDCl_3$) δ 171.29, 170.69, 158.01, 137.86, 129.01, 124.24, 119.71, 63.88, 36.98, 30.81, 28.08, 21.95, 21.01.

7-oxo-7-(Phenylamino)heptyl acetate (**26**). Yield: 72%, 191 mg. ^{1}H NMR (400 MHz, Chloroform-*d*) δ 8.15 (s, 1H), 7.55 – 7.48 (m, 2H), 7.29 – 7.21 (m, 1H), 7.09 – 7.00 (m, 1H), 4.00 (t, J = 6.7 Hz, 2H), 2.31 (t, J = 7.5 Hz, 2H), 2.01 (s, 3H), 1.68 (q, J = 7.5 Hz, 2H), 1.66 – 1.52 (m, 2H), 1.33 (h, J = 4.5, 3.7 Hz, 4H). ^{13}C NMR (101 MHz, $CDCl_3$) δ 171.84, 171.39, 138.22, 128.83, 124.06, 120.03, 119.98, 119.93, 77.47, 77.15, 76.83, 64.46, 37.38, 28.82, 28.40, 25.67, 25.49, 21.06, 20.98.

5-hydroxy-N-Phenylpentanamide (**27**). Yield: 61%, 100 mg. ^{1}H NMR (600 MHz, Chloroform-*d*) δ 7.49 (d, J = 7.9 Hz, 2H), 7.24 (d, J = 15.6 Hz, 2H), 7.04 (t, J = 7.4 Hz, 1H), 3.60 (t, J = 6.2 Hz, 2H), 3.28 (s, 1H), 2.35 (t, J = 7.4 Hz, 2H), 1.76 (p, J = 7.4 Hz, 2H), 1.62 – 1.53 (m, 2H). ^{13}C NMR (151 MHz, $CDCl_3$) δ 172.17, 138.10, 128.86, 124.17, 120.08, 77.28, 77.07, 76.86, 61.91, 36.91, 31.70, 21.94.

6-hydroxy-N-Phenylhexanamide (**28**). To a 20 mL scintillation vial with stir bar was added (**35**) (200 mg, 0.812 mmol) in 300 μL of methanol and 700 μL of 10% HCl. Vial was capped and stirred overnight at room temperature. Reaction was concentrated by rotary evaporation and purified by SiO_2 column chromatography to provide title compound as a white solid (270 mg, 99% yield). ^{1}H NMR (600 MHz, Chloroform-*d*) δ 8.28 (s, 1H), 7.52 – 7.47 (m, 2H), 7.23 (t, *J* = 7.9 Hz, 2H), 7.03 (t, *J* = 7.4 Hz, 1H), 3.56 (t, *J* = 6.5 Hz, 2H), 3.02 (s, 1H), 2.30 (t, *J* = 7.5 Hz, 2H), 1.67 (p, *J* = 7.6 Hz, 2H), 1.55 – 1.49 (m, 2H), 1.40 – 1.32 (m, 2H). ^{13}C NMR (151 MHz, $CDCl_3$) δ 171.83, 138.06, 128.94, 128.90, 128.87, 124.22, 124.17, 120.00, 119.96, 119.94, 77.29, 77.24, 77.22, 77.07, 77.02, 76.86, 76.82, 76.79, 62.42, 62.37, 37.46, 37.42, 37.39, 32.20, 32.15, 25.37, 25.32, 25.23, 25.18.

7-hydroxy-N-Phenylheptanamide (**29**). Yield: 91%, 115 mg. ^{1}H NMR (400 MHz, Chloroform-*d*) δ 8.09 (s, 1H), 7.52 (d, *J* = 7.9 Hz, 2H), 7.35 – 7.20 (m, 2H), 7.06 (t, *J* = 7.4 Hz, 1H), 3.59 (t, *J* = 6.5 Hz, 2H), 2.60 (s, 1H), 2.32 (t, *J* = 7.5 Hz, 2H), 1.77 – 1.61 (m, 2H), 1.52 (q, *J* = 6.7 Hz, 2H), 1.34 (p, *J* = 3.5 Hz, 4H). ^{13}C NMR (101 MHz, $cdcl_3$) δ 172.07, 138.13, 128.88, 124.14, 120.03, 119.99, 119.95, 77.40, 77.08, 76.76, 62.55, 37.39, 32.40, 28.87, 25.55, 25.41.

dibenzyl (5-oxo-5-(phenylamino)pentyl) phosphate (**30**). Yield: 65%, 85 mg. ^{1}H NMR (600 MHz, Chloroform-*d*) δ 8.12 (s, 1H), 7.59 – 7.53 (m, 2H), 7.34 – 7.25 (m, 10H), 7.09 – 7.03 (m, 1H), 5.09 – 4.96 (m, 4H), 4.01 (dt, *J* = 7.7, 6.1 Hz, 2H), 2.31 (t, *J* = 7.5 Hz, 2H), 1.81 – 1.71 (m, 2H), 1.71 – 1.61 (m, 2H). ^{13}C NMR (151 MHz, $cdcl_3$) δ 171.16, 138.40, 135.71, 135.67, 128.86, 128.61, 127.94, 123.88, 119.68, 77.25, 77.04, 76.83, 69.42, 69.38, 67.12, 67.08, 36.45, 29.25,

29.21, 21.66. ^{31}P NMR (243 MHz, cdcl$_3$) δ -0.91. HRMS (MALDI): m/z calculated for $C_{25}H_{28}NO_5P$ [M+H] 454.17051; observed 454.17703.

dibenzyl (6-oxo-6-(Phenylamino)hexyl) phosphate (**31**). Yield: 57%, 77 mg. ^{1}H NMR (600 MHz, Chloroform-*d*) δ 8.27 (s, 1H), 7.57 (d, *J* = 7.9 Hz, 2H), 7.37 – 7.22 (m, 10H), 7.04 (t, *J* = 7.5 Hz, 1H), 5.00 (dd, *J* = 8.4, 6.3 Hz, 4H), 3.95 (q, *J* = 6.8 Hz, 2H), 2.29 (t, *J* = 7.5 Hz, 2H), 1.70 – 1.63 (m, 2H), 1.63 – 1.54 (m, 2H), 1.35 (p, *J* = 7.8 Hz, 2H). ^{13}C NMR (151 MHz, cdcl$_3$) δ 171.53, 138.46, 135.76, 135.72, 128.83, 128.60, 128.59, 127.90, 123.89, 119.86, 77.33, 77.12, 76.91, 69.32, 69.28, 67.81, 67.76, 37.15, 29.84, 29.80, 24.97. ^{31}P NMR (243 MHz, cdcl$_3$) δ -1.20. HRMS (MALDI): m/z calculated for $C_{27}H_{32}NO_5P$ [M+1] 468.18616; observed 468.19424.

dibenzyl (7-oxo-7-(Phenylamino)heptyl) phosphate (**32**). Yield: 52%, 72mg. ^{1}H NMR (600 MHz, Chloroform-*d*) δ 7.90 (s, 1H), 7.57 – 7.54 (m, 2H), 7.36 – 7.29 (m, 10H), 7.27 (t, *J* = 7.9 Hz, 2H), 7.06 (t, *J* = 7.4 Hz, 1H), 5.10 – 4.93 (m, 4H), 3.96 (q, *J* = 6.6 Hz, 2H), 2.30 (t, *J* = 7.5 Hz, 2H), 1.67 (p, *J* = 7.2 Hz, 2H), 1.59 (q, *J* = 6.7 Hz, 2H), 1.33 (dd, *J* = 6.6, 3.4 Hz, 4H). ^{13}C NMR (151 MHz, cdcl$_3$) δ 171.53, 138.32, 135.83, 135.78, 128.87, 128.58, 128.54, 127.88, 123.94, 119.77, 77.25, 77.04, 76.83, 69.26, 69.22, 67.86, 67.83, 37.26, 29.86, 29.82, 29.68, 28.40, 25.27, 24.86. ^{31}P NMR (243 MHz, cdcl$_3$) δ -1.06. HRMS (MALDI): m/z calculated for $C_{27}H_{32}NO_5P$ [M+K] 520.20181; observed 520.16412.

6-((tert-butyldimethylsilyl)oxy)hexanoic acid (**34**). Compound **2** was synthesized using a previously published method. To a 25 mL round bottom flask with stir bar was added ε-Caprolatone (2.00 g, 17.52 mmol) and NaOH (2.10 g, 56.56 mmol, 3.0 equiv.) followed by 13

mL of H_2O. The solution was allowed to stir at room temperature overnight. Then the reaction was acidified to pH = 2 with 10% HCl and extracted with 3 x 25 mL of EtOAc. The combined organic layer was dried over $NaSO_4$ and dried in vacuo to provide product as a colorless oil (2.20 g, 95% yield) used in the next step without further purification. **TBDMS protection.** To a 100 mL round bottom flask with stir bar was added the opened lactone (2.20 g, 16.6 mmol), imidazole (2.26 g, 33.2 mmol, 2.0 equiv.), and tert-butyldimethylsilyl chloride (2.75 g, 18.62 mmol, 1.1 equiv.) and DMF (50 mL). The reaction was allowed to at room temperature, stir overnight and under argon. The reaction mixture was then diluted with 50 mL of EtOAc and washed with 3 x 50 mL of H_2O followed by 50 mL of brine. The organic layer was collected and treated with NaSO4 and dried *in vacuo*. The crude was purified by SiO_2 column chromatography to provide the title compounds as a colorless oil (1.90 g, 72% yield). ^{1}H NMR (300 MHz, Chloroform-*d*) δ 3.61 (t, *J* = 6.4 Hz, 2H), 2.43 – 2.31 (m, 3H), 1.72 – 1.59 (m, 3H), 1.59 – 1.47 (m, 2H), 1.45 – 1.32 (m, 2H), 0.88 (s, 9H), 0.04 (s, 6H). ^{13}C NMR (101 MHz, $CDCl_3$) δ 179.41, 77.31, 77.00, 76.68, 62.90, 33.95, 32.40, 26.00, 25.88, 25.68, 25.55, 25.32, 24.48, 18.33, -3.60, -3.65, -5.29, -5.33.

7-((tert-butyldimethylsilyl)oxy)-N-phenylheptanamide (**35**). Yield: 72%, 280 mg. ^{1}H NMR (400 MHz, Chloroform-*d*) δ 7.51 (d, *J* = 7.9 Hz, 2H), 7.31 (t, *J* = 7.9 Hz, 2H), 7.25 – 7.22 (m, 1H), 7.09 (t, *J* = 7.4 Hz, 1H), 3.61 (t, *J* = 6.4 Hz, 2H), 2.36 (t, *J* = 7.6 Hz, 2H), 2.17 (s, 2H), 1.75 (p, *J* = 7.6 Hz, 2H), 1.61 – 1.51 (m, 2H), 1.48 – 1.36 (m, 2H), 0.88 (s, 9H), 0.04 (s, 9H). ^{13}C NMR (101 MHz, $CDCl_3$) δ 171.27, 137.93, 128.97, 124.16, 119.76, 77.34, 77.02, 76.70, 62.98, 37.80, 32.55, 25.99, 25.94, 25.54, 25.43, 18.36, -5.28.

O,O-bis(2-Cyanoethyl) O-(6-oxo-6-(phenylamino)hexyl) phosphorothioate (**36**). To a flame dried 5 mL round bottom flask with stir bar under argon atmosphere was added Bis(2-cyanoethyl)-*N,N*-diisopropylphosphoramidite, (100 mg, 0.369 mmol, 1.0 equiv.) in acetonitrile (2 mL). Solution was cooled to 0 ^{o}C, then 5-(ethyl)-1H-tetrazole (54.4 mg, 0.554 mmole, 1.5 equiv.) was added and the mixture was allowed to stir for 30 min. A solution of alcohol (**28**) (115 mg, 0.554 mmol, 1.5 equiv.) in acetonitrile (1 mL) was added dropwise to the phosphoramidite. After 2 hours cyclo-octasulfur (478 mg, 1.85 mmol, 5 equiv.) was added then reaction was stirred overnight. Mixture was filtered, concentrated and residue was purified by column chromatography to provide title compounds as a colorless oil (79 mg, 52% yield). ^{1}H NMR (400 MHz, Chloroform-*d*) δ 7.66 (s, 1H), 7.58 – 7.48 (m, 2H), 7.30 (t, *J* = 7.9 Hz, 2H), 7.09 (t, *J* = 7.3 Hz, 1H), 4.25 (dt, *J* = 9.7, 6.0 Hz, 4H), 4.14 (dt, *J* = 9.9, 6.3 Hz, 2H), 2.75 (td, *J* = 6.1, 0.8 Hz, 4H), 2.38 (t, *J* = 7.4 Hz, 2H), 1.75 (ddd, *J* = 15.0, 9.1, 7.0 Hz, 4H), 1.49 (td, *J* = 8.2, 4.1 Hz, 2H). ^{31}P NMR (162 MHz, $CDCl_3$) δ 67.16. HRMS (MALDI): m/z calculated for $C_{18}H_{24}N_3O_4PS$ [M+H] 410.12251; observed 410.13107.

In Vitro Assay.

Compounds (**1-7**) were dissolved in milliQ water to make 100 mM stock solutions and SAHA were diluted with milliQ water to make 25 mM stock solutions. All compound stock solutions were kept frozen at -80 ^{o}C until assay was performed. Optimal receptor/inhibitor binding incubation time was found by adding 40 μL of buffer, 5 μL of cell lysate, HDAC3 or HDAC8 enzyme, and 5 μL of **2** (100 μM final concentration) to a 96 well plate. Plate was incubated for 5 min, 1 hr, 2 hr, 4 hr and 8 hr at 30 ^{o}C. Then 50 μL of substrate (0.1 mM final concentration) was added and the mixture was incubated at 30 ^{o}C for 30 min. Next, 10 uL of developer solution was

then added followed by further incubation for 10 min at 25 ^{o}C. The enzyme activities was measured based on the fluorescence intensity at excitation 350-380 nm and emission 440-480 nm in a plate reader. A control set of data without the presence of inhibitor was acquired using 45 μL of buffer. Inhibitors **1-7** were screened using a commercially available *In Situ* Histone Deacetylase Activity Fluorometric Assay Kit. The manufacturer's suggested protocol was followed with the only divergence being the 8 hr incubation time.
